# The GAR/RGG motif defines a family of nuclear alarmins

**DOI:** 10.1038/s41419-021-03766-w

**Published:** 2021-05-12

**Authors:** Shan Wu, Boon Heng Dennis Teo, Seng Yin Kelly Wee, Junjie Chen, Jinhua Lu

**Affiliations:** 1 Department of Microbiology and Immunology, Yong Loo Lin School of Medicine, National University of Singapore, Blk MD4, 5 Science Drive 2, Singapore, 117545 Singapore; 2 Immunology Translational Research Programme, Yong Loo Lin School of Medicine, National University of Singapore, 28 Medical Drive, Singapore, 117456 Singapore

**Keywords:** Cell death and immune response, Systemic lupus erythematosus

## Abstract

The nucleus is the target of autoantibodies in many diseases, which suggests intrinsic nuclear adjuvants that confer its high autoimmunogenicity. Nucleolin (NCL) is one abundant nucleolar autoantigen in systemic lupus erythematosus (SLE) patients and, in lupus-prone mice, it elicits autoantibodies early. With purified NCL, we observed that it was a potent alarmin that activated monocytes, macrophages and dendritic cells and it was a ligand for TLR2 and TLR4. NCL released by necrotic cells also exhibited alarmin activity. The NCL alarmin activity resides in its glycine/arginine-rich (GAR/RGG) motif and can be displayed by synthetic GAR/RGG peptides. Two more GAR/RGG-containing nucleolar proteins, fibrillarin (FBRL) and GAR1, were also confirmed to be novel alarmins. Therefore, the GAR/RGG alarmin motif predicts a family of nucleolar alarmins. The apparent prevalence of nucleolar alarmins suggests their positive contribution to tissue homeostasis by inducing self-limiting tissue inflammation with autoimmunity only occurring when surveillance is broken down.

## Introduction

Despite of the central tolerance mechanisms, polyreactive B cells remain common in the naive repertoire as potential origins of pathogenic autoantibodies^1,2^. Nuclear antigens are frequently targeted by these autoantibodies^3^. In systemic lupus erythematosus (SLE) patients, pathogenic IgG autoantibodies originate from polyreactive B cells and, in mice, polyreactive B cells induce autoreactive germinal centers to activate autoreactive B cells of broader specificity^4,5^. Aberrant monospecific B-cell somatic hypermutation also gives rise to autoreactive B cells^6^.

Among patients with antinuclear autoantibodies (ANA), 10–15% develop autoantibodies that react predominantly with the nucleolus^7–9^. Nucleolar proteins mostly function in ribosome biogenesis and their high autoimmunogenicity are not explained^10,11^. Some ribonucleoproteins (RNPs) contain pro-inflammatory RNA components, which can confer immunogenicity to the protein components^12,13^. The nucleoli contain abundant RNPs but pro-inflammatory nucleolar RNA or proteins have not been reported^11^. Nucleolin (NCL) is an abundant nucleolar protein with multiple RNA-binding motifs^10,14,15^. It is not a known RNP component but it is a major autoantigen in a subgroup of SLE patients^16^. In lupus-prone mice, NCL-specific autoantibodies appeared early before other autoantibodies, suggesting an early pathogenic role^17^.

Besides the nucleolus, NCL is also a nucleocytoplasmic shuttling protein and participates in nuclear import and export^18,19^. It localizes on the cell surface^20,21^, and could be secreted^22^. Cell surface NCL interacts with diverse proteins^20,21^, such as P- and L-selectins^22,23^, and the respiratory syncytial virus^24^, and can mediate protein endocytosis^20,21^. NCL is targeted to the cell surface through cytoskeleton or small vesicles^22,25^. How cell surface NCL is related to autoimmunity is unclear.

Necrotic cells release nuclear alarmins that can cause tissue inflammation and also release autoantigens that activate B cells^26–28^. Interestingly, in necrotic cells, the highly autoimmunogenic nucleoli are the dominant targets of complement protein C1q^29^. C1q binds to the nucleolar region where NCL localizes^29^. Binding of C1q to nucleoli activates C1q-associated proteases, which effectively degrade nucleolar proteins including NCL^29,30^. This adds C1q and its associated proteases to the intracellular and extracellular surveillance mechanisms, which function to limit necrotic tissue inflammation and autoimmunity^31–34^. The importance of this C1q-mediated surveillance is reflected by deficiency of C1q or C1q-associated proteases causing ANA production and severe SLE pathogenesis^35^. With these observations, we asked whether, like high mobility group box 1 (HMGB1)^36^, NCL possesses intrinsic alarmin activity that confers nucleolar immunogenicity.

## Materials and methods

### Antibodies and reagents

Rabbit antibodies against NCL (ab22758) and HMGB1 (ab67281) and a mouse TLR2-blocking antibody (ab9100) were obtained from Abcam (Cambridge, UK). A mouse anti-NCL antibody (sc-8031) was purchased from Santa Cruz Biotechnology, Inc (Dallas, TX). Lipopolysaccharide (LPS) and mouse IgG1 (M9269) were from Sigma-Aldrich (St. Louis, MO). Recombinant human TLR2-10xHis (2616-TR) was from R&D Systems (Mineapolis, MN). Anti-HA-agarose was from ThermoFisher Scientific (Walthem, MA). A TLR4-blocking mouse antibody (Mabg-htlr4), a TLR5-blocking human antibody (Maba-htlr5), an interleukin (IL)-1β-blocking mouse antibody, lipoteichoic acid (LTA, tlrl-slta), flagellin (tlrl-stfla), and poly I:C (tlrl-picw), were from InvivoGen (San Diego, CA).

### Protein purification

TxNE (Triton X-100-depleted Nucleus Extract)^37^, was used to purify NCL and HMGB1. Briefly, 60 μg of NCL or HMGB1 antibodies or non-immune mouse IgG1 were bound to protein G-Sepharose (600 μl) (GE Healthcare, Chicago, IL). After incubation for 30 min in PBS containing dimethyl pimelimidate (0.2 M) and triethanolamine (pH 8–9), beads were washed and blocked in PBS containing ethanolamine (50 mM), pre-eluted with 0.1 M glycine (pH 2.5), equilibrated in TBS (50 mM Tris, pH 7.4 and 150 mM NaCl), and incubated overnight with TxNE. After washing in a wash buffer (0.25 M sucrose, 10 mM Tris, pH 7.4, 250 mM NaCl, 3.3 mM CaCl_2_, 0.1% (v/v) Tween 20), proteins were eluted using 0.1 M glycine (pH 2.5), collecting 10 × 0.3 ml fractions, and dialyzed against PBS.

NCL, FBRL, and GAR1 sequences were cloned in the pcDNA3.1 plasmid with C-terminal HA (Figs. 3A and 5), to transfect the human embryonic kidney 293T cells (ATCC)^38^. After culturing for 48 h in Dulbecco’s modified Eagle medium containing 10% (v/v) heat-inactivated fetal bovine serum, l-glutamine (2 mM), and penicillin/streptomycin (100 units/ml) in the presence of 5% CO_2_, cells were homogenized to isolate cytoplasm and TxNE^37^, which were combined to incubate overnight with anti-HA-Agarose (0.3 ml). Beads were washed with the wash buffer (30 ml), and eluted using 3.5 M MgCl_2_, collecting 10 × 0.3 ml fractions. Proteins were dialyzed in PBS and measured using the Bradford reagent (Bio-Rad, Hercules, CA). Endotoxin was monitored using an LAL Endotoxin Assay (GenScript, Piscataway, NJ) and contamination by RNA and other TLR ligands were excluded based on RNase and trypsin digestion (Supplementary Fig. S1).

### Sodium dodecyl sulphate–polyacrylamide gel electrophoresis (SDS-PAGE) and western blotting

Protein samples were boiled in the presence of dithiothreitol (10 mM) and, after separation on 12.5% (w/v) SDS-PAGE gels, electro-blotted on PVDF membranes. Blots were blocked for 1 h with 5% (w/v) non-fat milk in TBS-T (50 mM Tris, pH 7.4, 150 mM NaCl and 0.1% (v/v) Tween 20), incubated overnight with antibodies, and after washing incubated for 1 h with HRP-conjugated secondary antibodies. Blots were developed using the Pierce SuperSignal West Pico chemiluminescent substrate (ThermoFisher Scientific).

### Blood cell isolation and culturing

Buffy coats were obtained from the Singapore Health Sciences Authority Blood Transfusion Services from healthy blood donors with institutional ethics approval. Peripheral blood mononuclear cells (PBMC) were isolated using Ficoll-Paque (GE Healthcare). PBMC (1 × 10^7^/ml) were re-suspended in RPMI medium containing 5% (v/v) bovine calf serum (HyClone) and cultured for 1 h to harvest adherent monocytes, which were re-suspended at 1.5 × 10^6^ cells/ml. Monocytes were cultured with macrophage colony-stimulating factor (20 ng/ml) to generate macrophages, and dendritic cells (DC) were cultured using granulocyte-macrophage colony-stimulating factor (20 ng/ml) and IL-4 (40 ng/ml)^38^.

### Cell activation

Purified proteins (40 μg/ml) were coated for 12 h in 96-well plates (50 μl/well). PBMC (3 × 10^6^/ml), monocytes (1 × 10^6^/ml), macrophages (0.5 × 10^6^/ml) or DC (0.5 × 10^6^/ml) were re-suspended in the Macrophage Serum-free medium (ThermoFisher Scentific), containing penicillin and streptomycin, and cultured for 24 h in protein-coated plates (100 μl/well). Cells are also stimulated with TLR ligands: LPS (0.5 μg/ml for DC and macrophages and 10 ng/ml for PBMC and monocytes), flagellin (1 μg/ml), and lipoteichoic acid (LTA, 10 μg/ml). Tumor necrosis factor alpha (TNFα) and IL-1β production was determined by ELISA (Invitrogen). PBMC were also pre-incubated for 1 h with the MyD88 inhibitor st-2825 (MedChemExpress) or the Caspase-1 inhibitor Ac-YVAD (InvivoGen) before stimulation. The st-2825 and Ac-YVAD concentrations were optimized based on inhibition and cytotoxicity as determined using the CellTiter 96® AQueous One Solution Cell Proliferation Assay (Promega Co. Madison, WI). All peptides were dissolved in PBS. Monocytes were also pre-blocked with TLR2, TLR4, or TLR5 antibodies for 1 h before stimulation with TLR ligands, NCL or HMGB1.

### NF-κB luciferase assay

TLR activation was measured using an NFκB-based luciferase assay (Promega). Firefly luciferase was expressed under the NF-κB gene promoter (p5xNFκB-Luc, Stratagene, San Diego, CA) and *Renilla* luciferase was expressed under the CMV promoter (pRL-CMV, Promega)^39^. 293T cells were transfected in 24-well plates with these luciferase vectors and co-transfected with TLR and co-receptor vectors^39^, each at 0.1 μg/well using TurboFect (ThermoFisher Scientific). After 24 h, cells were harvested and stimulated in 96-well plates with purified proteins, synthetic peptides, or microbial TLR ligands. NFκB-mediated firefly luciferase activity was measured and normalized to *Renilla* luciferase activity in each well and expressed as relative NFκB activation.

### TLR2-binding assay

Ninety-six-well plates were coated with purified proteins (1.0 μg/well) and blocked for 1 h with PBS containing 1% (w/v) bovine serum albumin (PBS-BSA). TLR2 was diluted (0.375 to 6 μg/ml) to incubate with the plates overnight at 4 °C and bound TLR2 was detected by incubating for 1 h with a mouse anti-His antibody (Sigma-Aldrich) and then 30 min with an HRP-conjugated secondary antibody (DAKO, Glostrup, Denmark). TLR2 was also coated (2 μg/ml) to incubate with NCL, NCL-HA, or the NCL peptides. Bound NCL was detected using a rabbit anti-NCL antibody and bound NCL-HA and its mutants were detected using a mouse anti-HA antibody (1 μg/ml), followed by HRP-conjugated secondary antibodies. Plate-coated TLR2 was also pre-incubated with mouse TLR2 or TLR4-blocking antibodies (5 μg/ml) before incubation with NCL or NCL-HA, and bound NCL or NCL-HA was detected using a rabbit anti-NCL antibody. Coated TLR2 was incubated with biotin-Ahx-tagged peptides and the bound peptides were detected with streptavidin-HRP. Plates were all developed using the 3, 3′, 5, 5′-Tetramethylbenzidine (TMB) substrate solution (ThermoFisher Scientific).

### Statistical analysis

All experiments were performed in triplicates. Data were representative of three independent experiments and presented as mean ± SD. Statistics was performed by one-way ANOVA or student *t*-test to compare two groups using the Prism software (GraphPad Prism 7). *p*-values were indicated by *p < 0.05, **p < 0.01, ***p < 0.001, ****p < 0.0001. *p* < 0.05 was considered statistically significant.

## Results

### NCL activates leukocytes

**Fig. 1:**
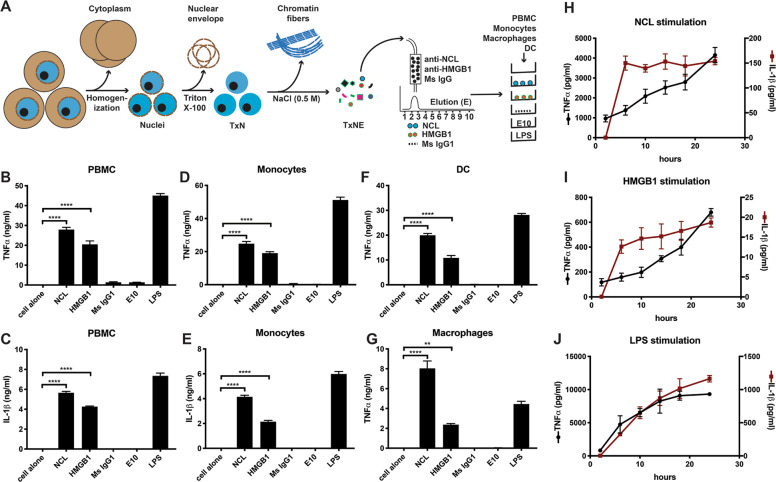
NCL activates PBMC, monocytes, macrophages, and dendritic cells (DC). **A** NCL and HMGB1 isolation and leukocyte stimulation. HeLa cell nuclei were first extracted in 1% (v/v) Triton X-100 (TxN) and proteins were extracted from TxN using 0.5 M NaCl as TxNE from which NCL and HMGB1 were affinity-purified. As a control, TxNE was also applied on immobilized mouse IgG1 (Ms IgG1). Elution from each column (fractions 2 and 3) were pooled. Fraction 10 lacked detectable proteins and were combined as another control (E10). Pooled NCL, HMGB1 and controls were coated on the plate to stimulate PBMC, monocytes, macrophages and DC. LPS (0.5 μg/ml for DC and macrophages and 10 ng/ml for PBMC and monocytes) was used as a positive control. **B**, **C** TNFα and IL-1β induction from PBMC. **D**, **E** TNFα and IL-1β induction from monocytes. **F**, **G** TNFα induction from macrophages and DC. **H**, **I**, **J** Kinetics of TNFα and IL-1β induction from PBMC (2.5, 5, 10, 14, 18, and 24 h). TNFα and IL-1β were measured by ELISA. Triplicate experiments were performed to obtain data as mean ± SD. Data was analyzed by one-way ANOVA. *p < 0.05, **p < 0.01, ****p < 0.0001.

Purified NCL was coated to stimulate PBMC, using HMGB1 as a control (Fig. 1A)^36,40^. TxNE was also applied to immobilized non-immune mouse IgG1 (Ms IgG1) and equivalent elution was used as a control. The protein-free 10^th^ fractions eluted from the affinity resins (E10) were also combined as a control. Plate-coated NCL and HMGB1 induced TNFα and IL-1β from PBMC (Fig. 1B, C), and from monocytes (Fig. 1D, E). The E10 fraction and IgG1 elution showed no cytokine induction (Fig. 1B, E). NCL also activated macrophages and DC (Fig. 1F, G). It consistently induces more cytokines than HMGB1, which is a ligand for TLR2, TLR4, and TLR5^40,41^. Although NCL binds to RNA, RNase digestion of plate-coated NCL showed no impairment in PBMC activation (Supplementary Fig. S1B), but trypsin digestion abolished NCL activation of PBMC (Supplementary Fig. S1C). Trypsin-digested TLR ligands, i.e., LPS, LTA and poly I:C, showed no reduction in PBMC activation.

NCL and HMGB1 were compared with LPS in TNFα and IL-1β induction (Fig. 1H–J). Both NCL and HMGB1 induced an early IL-1β surge and a late TNFα surge (Fig. 1H, I). TNFα production was partially blocked by an IL-1β-specific antibody, suggesting autocrine IL-1β stimulation of monocytes (Supplementary Fig. S2). LPS did not induced the type of early IL-1β or late TNFα surge, which were observed with NCL and HMGB1 stimulation (Fig. 1J). The similar cytokine induction by NCL and HMGB1 suggests their activation of similar receptors which, for HMGB1, are TLRs.

### TLR2 and TLR4 are NCL receptors

**Fig. 2:**
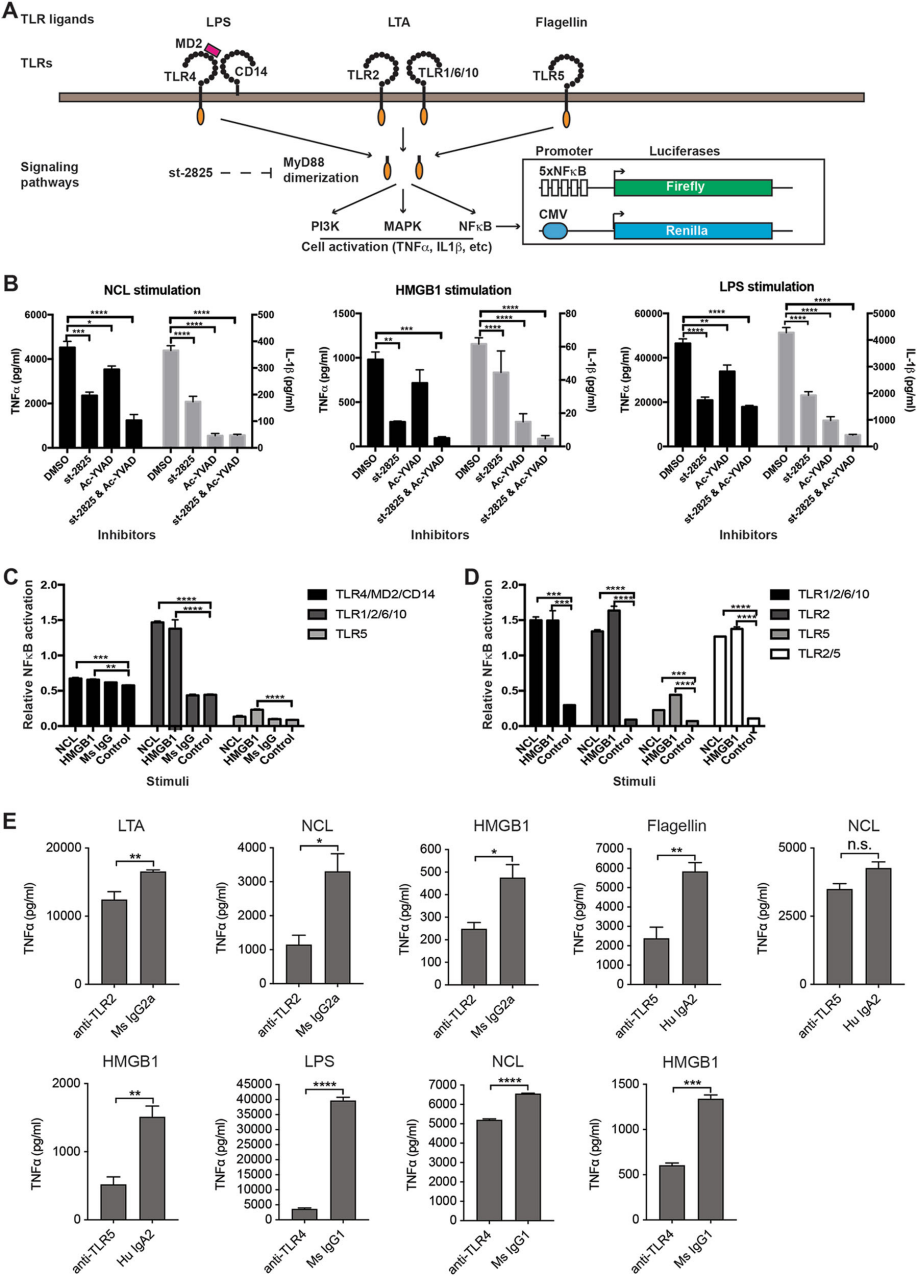
TLR2 and TLR4 are NCL receptors. **A** The NFκB luciferase assay. TLR4, MD2 and CD14 collectively respond to LPS. TLR2 often dimerizes with TLR1, TLR6 or TLR10 to respond to its ligands. After ligand binding, most TLRs recruit MyD88 to activate NF-κB and other signaling pathways. In this assay, TLRs and two luciferase reporters, i.e., p5xNFκB-Luc and pRL-CMV, were co-expressed in 293T cells. After TLR ligand stimulation, NFκB-directed luciferase expression was measured. **B** Roles of MyD88 and caspase-1 in PBMC response to NCL. PBMC were pre-treated with the MyD88 inhibitor st-2825 (30 μM), the caspase-1 inhibitor Ac-YVAD (10 μM), or both. Cells were then stimulated with plate-coated NCL or, as controls, plate-coated HMGB1 (40 μg/ml) or soluble LPS (10 ng/ml). TNFα and IL-1β were measured by ELISA. **C**, **D** NCL and HMGB1 activation of TLR signaling. TLR2, TLR4 or TLR5 was each co-expressed with their co-receptors and the luciferase reporters and then stimulated with coated NCL or HMGB1 (40 μg/ml) or soluble LPS (0.5 μg/ml). TLR activation was measured based on NFκB-directed firefly luciferase expression, which was normalized to CMV-directed *Renilla* luciferase expression in each well. Triplicate experiments were performed to obtain data as mean ± SD, which were analyzed by one-way ANOVA. *p < 0.05, **p < 0.01, ***p < 0.001, ****p < 0.0001. **E** Roles of TLR2, TLR4 and TLR5 in monocyte response to NCL, HMGB1 and LPS. Monocytes were pre-incubated with TLR2, TLR4, or TLR5-blocking antibodies before stimulation with coated NCL, coated HMGB1 or soluble microbial ligands for TLR2 (LTA,10 μg/ml), TLR4 (LPS, 10 ng/ml), or TLR5 (flagellin, 1 μg/ml). Triplicate experiments were performed and TNFα production was measured. Data were analyzed by student *t*-test. *p < 0.05, **p < 0.01, ***p < 0.001, ****p < 0.0001.

MyD88-mediated TLR signalling is inhibited by st-2825 (Fig. 2A)^42^. At 30 μM, st-2825 effectively inhibited LPS induction of cytokines without causing significant monocyte cytotoxicity (Supplementary Fig. S3A). The caspase-1 inhibitor Ac-YVAD was similarly titrated and was used at 10 μM (Supplementary Fig. S3B). st-2825 significantly inhibited NCL, HMGB1 and LPS induction of both TNFα and IL-1β production (Fig. 2B). Ac-YVAD completely abolished IL-1β induction by these stimuli and also partially inhibited their induction of TNFα (Fig. 2B). The latter was likely secondary to inhibiting autocrine IL-1β stimulation (Supplementary Fig. S2).

NCL activation of TLRs was examined after TLRs expression in 293T cells (Fig. 2A)^39^. TLRs were expressed in 4 groups (i.e., TLR2/1/6/10, TLR4/CD14/MD2, TLR5, and TLR3/7/8/9), and co-transfected with the p5xNFkB-Luc and pRL-CMV luciferase reporter vectors (Fig. 2A). TLR3/7/8/9 and TLR5 did not respond to NCL stimulation (Supplementary Fig. S4). TLR4/CD14/MD2 exhibited high-NFκB autoactivation^39^, but NCL induced significant further activation (Fig. 2C). TLR2/1/6/10 showed the most prominent response to NCL (Fig. 2C). HMGB1 activated TLR2/1/6/10, TLR4/MD2/CD14, and TLR5 (Fig. 2C)^40,41^. TLR2 alone was sufficient to respond to NCL and was not affected by TLR5 co-expression (Fig. 2D). Overall, TLR2 and probably TLR4 are NCL receptors.

To understand TLR2 response to NCL on monocytes, cells were pre-blocked with TLR2-, TLR4- or TLR5-blocking antibodies before NCL, HMGB1 or microbial ligands stimulation, i.e., lipoteichoic acid (LTA, TLR2), LPS (TLR4), and flagellin (TLR5) (Fig. 2E). All three antibodies significantly inhibited monocyte response to HMGB1 but monocyte response to NCL was only inhibited by the TLR2 and TLR4 antibodies (Fig. 2E), which agrees with the luciferase data (Fig. 2D).

### The alarmin activity resides in the NCL GAR/RGG motif

**Fig. 3:**
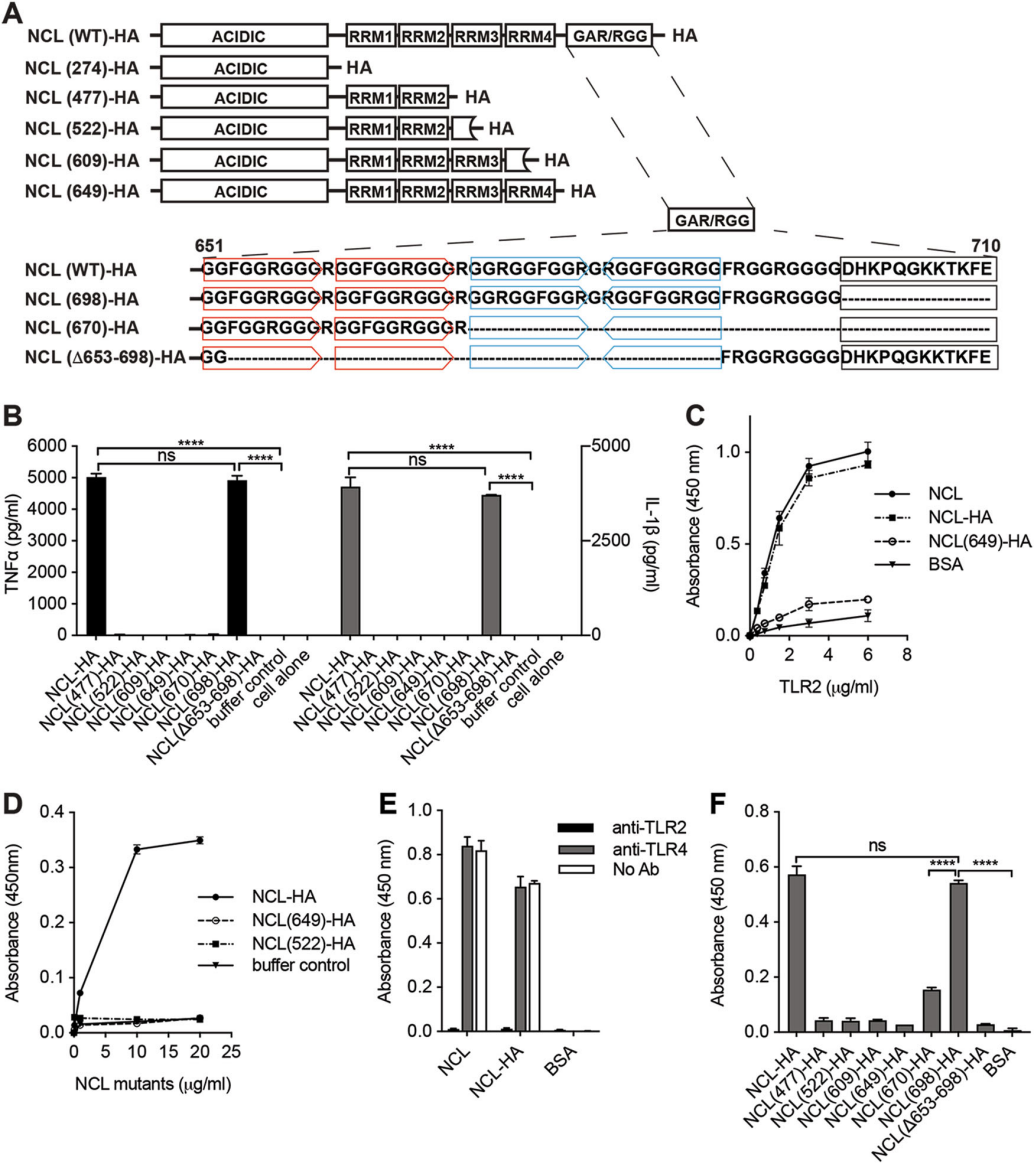
The NCL alarmin activity resides in its GAR/RGG motif. **A** Recombinant NCL and its mutants. NCL mutants were generated by C-terminal deletion and the new C-termini are bracketed (i.e., 274, 477, 522, 609, 649, 670 and 698). Two head-to-head repeats in the GAR/RGG motif were highlighted in red boxes and two head-to-tail repeats were identified in cyan boxes. Deletion of these four repeats yields another mutant (Δ653-698). Recombinant NCL and its mutants were expressed in 293T cells with C-terminal HA tags. **B** Monocytes were stimulated with coated NCL-HA and its mutants (40 μg/ml) for 24 h to measure TNFα and IL-1β production. Triplicate experiments were performed to obtain data as mean ± SD. Data were analyzed by one-way ANOVA. **C** NCL, NCL-HA, NCL(649)-HA, and BSA (10 μg/ml) were coated to incubate with TLR2-10xHis (0.375–6.0 μg/ml). Bound TLR2 was detected using a mouse anti-His antibody (2.6 μg/ml). **D** TLR2 (2 μg/ml) was coated to incubate with NCL-HA, NCL(649)-HA, or NCL(522)-HA (0–20 μg/ml). Bound NCL-HA and its mutants were detected using a mouse anti-HA antibody (1 μg/ml). **E** TLR2 (2 μg/ml) was coated, pre-incubated with TLR2 or TLR4-blocking antibodies (5 μg/ml), and incubated with NCL, NCL-HA or BSA (10 μg/ml). Bound proteins were detected using a rabbit anti-NCL antibody (1 μg/ml). **F** Binding of NCL-HA and its mutants to TLR2. NCL-HA, its mutants, and control BSA were plate-coated (10 μg/ml) to incubate with TLR2 (2 μg/ml). Bound TLR2 was detected using the mouse anti-His antibody. In these experiments, HRP-conjugated secondary antibodies were used. Triplicate experiments were performed to obtain data as mean ± SD. Data was analyzed by one-way ANOVA. *p < 0.05, ***p < 0.001, ****p < 0.0001. n.s., not significant.

NCL contains an N-terminal acidic domain (277 AA), four RNA recognition motifs (RRM1-4) (375 AA)^43^, a GAR/RGG motif (48 AA)^44^, and a short C-terminal tail (12 AA) (Fig. 3A). The GAR motifs generally also bind to RNA^43,44^. NCL was expressed with an HA tag (NCL-HA) to generate seven C-terminal deletion mutants. Six mutants lost monocyte activation (Fig. 3A, B). The only stimulatory NCL mutant, NCL(698)-HA, only lacked 12 AA from the C-terminus and deleting another 28 AA into the upstream GAR/RGG motif yielded an alarmin-inactive mutant, NCL(670)-HA (Fig. 3A, B). This suggests alarmin activity inside the 48-AA GAR/RGG motif. Deleting 37-AA inside the GAR/RGG motif indeed also yielded an alarmin-inactive NCL(Δ653-698)-HA mutant (Fig. 3A, B).

### TLR2 binds to the NCL GAR/RGG motif

To determine whether TLR2 binds to the NCL GAR/RGG motif, purified NCL, NCL-HA and NCL(649)-HA were coated to incubate with TLR2. TLR2 bound to NCL and NCL-HA in dose-dependent and saturable manners but it lacked binding to NCL(649)-HA (Fig. 3C). When TLR2 was coated to incubate with NCL-HA and its mutants, NCL(649)-HA and NCL(522)-HA, NCL-HA but not the two mutants bound to TLR2 (Fig. 3D). If coated TLR2 was pre-blocked with an anti-TLR2 antibody (Fig. 2E), it no longer bound to NCL or NCL-HA (Fig. 3E). The TLR4-blocking antibody showed no inhibition (Fig. 3E), suggesting that the NCL GAR/RGG motif binds to monocyte surface TLR2 to cause its activation.

Other NCL-HA mutants were also coated and TLR2 bound to the alarmin-active NCL(698)-HA but not the alarmin-inactive NCL-HA mutants (Fig. 3A, F). NCL(670)-HA contained a residual GAR/RGG region but it only showed weak TLR2 binding (Fig. 3A, F). Therefore, TLR2 only binds to the GAR/RGG motif on NCL. We then asked whether synthetic GAR/RGG peptides would also bind to and activate TLR2.

### Identification of two alarmin peptides in the NCL GAR/RGG motif

**Fig. 4:**
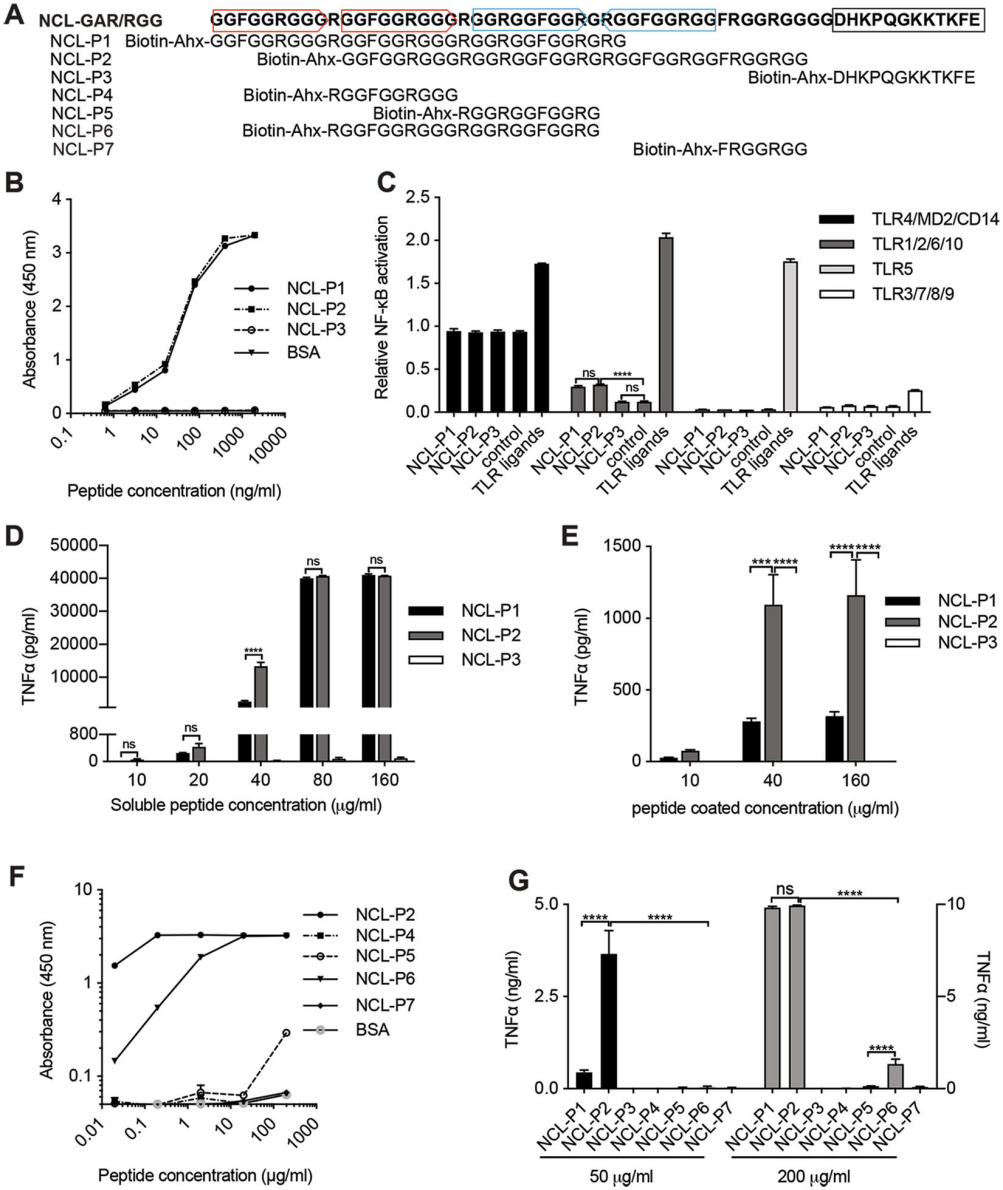
NCL GAR/RGG peptides bind to TLR2 and activate monocytes. **A** Peptides were synthesized with N-terminal biotin-Ahx: six peptides corresponded to the GAR/RGG motif and one peptide represented the NCL C-terminal tail (NCL-P3). **B** TLR2 was coated (2 μg/ml) to incubate with NCL-P1, NCL-P2 or NCL-P3 and bound peptides were detected using streptavidin-HRP. **C** In 293T cells, TLR4/CD14/MD2, TLR2/1/6/10, TLR5 or TLR3/7/8/9 were co-expressed with the firefly and *Renilla* luciferase reporters. After 24 h, cells were stimulated with soluble NCL peptides (200 μg/ml). TLR activation was determined by measuring NF-κB activation. TLR ligands: LPS (TLR4, 0.5 μg/ml), LTA (TLR2, 10 μg/ml), flagellin (TLR5, 1 μg/ml), and PolyI:C (TLR3, 50 μg/ml). **D** Monocytes were stimulated with the NCL peptides and TNFα production was measured by ELISA. **E** Peptides were coated at 10, 40, or 160 μg/ml to stimulate monocytes and TNFα production was measured by ELISA. **F** TLR2 was coated (2 μg/ml) to incubate with NCL-P4, NCL-P5, NCL-P6, NCL-P7 for which NCL-P2 and BSA were used as controls. Bound peptides were detected using streptavidin-HRP. **G** Monocytes were stimulated with the NCL peptides (50 or 200 μg/ml). TNFα production was measured by ELISA. Triplicate experiments were performed. Data were presented as mean ± SD and analyzed by one-way ANOVA. ****p < 0.0001. n.s. not significant.

The NCL GAR/RGG motif contains two head-to-tail repeats (651GGFGGRGGGRggfggrgggr670), two tail-to-tail repeats (671GGRGGFGGRgRGGFGGRGG689), and a non-repetitive C-terminal region (690FRGGRGGGG698) (Figs. 3A and 4A). Two overlapping peptides were synthesized: NCL-P1 (32 AA) and NCL-P2 (36 AA) (Fig. 4A). The C-terminal 12 AA was synthesized as a control peptide (NCL-P3) (Fig. 4A). All peptides contained N-terminal biotin-Ahx tags and were incubated with plate-coated TLR2.

NCL-P1 and NCL-P2, but not NCL-P3, exhibited dose-dependent and saturable binding to TLR2 (Fig. 4B). NCL-P1 and NCL-P2 also activated TLR2-mediated NFκB signaling and induced cytokines from monocytes (Fig. 4C, D). At intermediate peptide concentrations (10-40 μg/ml), NCL-P2 induced ~10x more TNFα than NCL-P1 (Fig. 4D). In the plate-coated form, NCL-P2 also induced more cytokines than NCL-P1 (Fig. 4E). Therefore, NCL-P2 is a stronger TLR2 ligand than NCL-P1. It was observed that soluble NCL-P1 and NCL-P2 were stronger alarmins than their immobilized forms (Fig. 4D, E). In contrast, coated NCL induced more cytokines than soluble NCL (Supplementary Fig. S5). We suspect that coated NCL could confer multivalent TLR2 stimulation but coating NCL peptides might mask their TLR2 binding sites.

### Shorter GAR/RGG peptides lose their alarmin activities

Since both NCL-P1 and NCL-P2 have alarmin activities, we suspected that their 21-AA overlapping region contained the TLR2 ligand and synthesized this region as NCL-P6 (Fig. 4A). NCL-P4 and NCL-P5 were synthesized as the N- and C-terminal halves of NCL-P6 (Fig. 4A). The GAR/RGG region outside NCL-P1/NCL-P2 was synthesized as NCL-P7 (Fig. 4A). NCL-P6 only bound to TLR2 weakly and NCL-P4, NCL-5 and NCL-P7 lacked binding (Fig. 4F). Therefore, for the NCL GAR/RGG motif to bind to TLR2, there is a length requirement.

### The GAR/RGG-containing fibrillarin (FBRL) and the H/ACA ribonucleoprotein complex subunit 1 (GAR1) are also alarmins

**Fig. 5:**
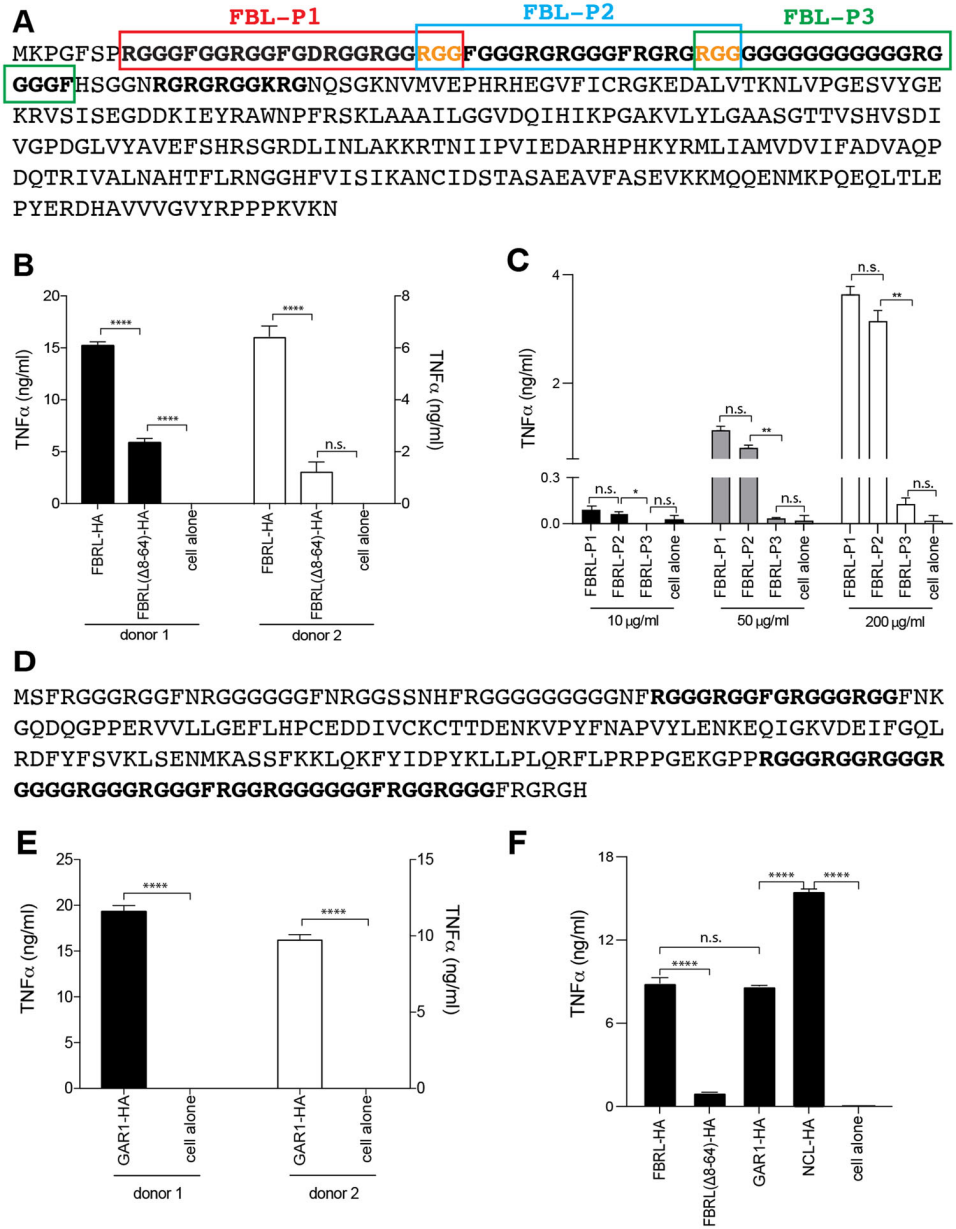
Fibrillarin (FBRL) and GAR1 are nucleolar alarmins. **A** FBRL sequence showing a GAR/RGG and a GAR/RG motif. Three peptides were synthesized as indicated and overlapping residues were highlighted (orange font). The GAR/RGG motif was deleted to generate the FBRL(Δ8-64)-HA mutant. **B** FBRL-HA and FBRL(Δ8-64)-HA were purified and plate-coated (40 μg/ml) to stimulate PBMC for 24 h and TNFα production was measured by ELISA. **C** PBMC were stimulated for 24 h with the FBRL peptides (10, 50, or 200 μg/ml) and TNFα production was determined by ELISA. **D** GAR1 sequence showing two GAR/RGG motifs. **E** GAR1-HA (40 μg/ml) was purified and coated to stimulate PBMC and TNFα production was determined by ELISA. **F** Comparison of FBRL, GAR1, and NCL in alarmin activities. The purified proteins were plate-coated (40 μg/ml) to stimulate PBMC for 24 h. TNFα production was determined by ELISA. Cell controls, no stimulation. Triplicate experiments were performed to obtain data as mean ± SD. Data was analyzed by one-way ANOVA *p < 0.05, ****p < 0.0001, n.s., not significant.

Among the GAR motifs, GAR/RGG only represents a subgroup^44^. The nucleolar proteins fibrillarin (FBRL) and GAR1 contain characteristic GAR/RGG motifs (Fig. 5). FBRL was expressed as FBRL-HA and it strongly activated PBMC (Fig. 5B). This alarmin activity largely diminished after its GAR/RGG motif was deleted (Fig. 5B). Three peptides were synthesized based on the FBRL GAR/RGG region (Fig. 5A). FBRL-P1 and FBRL-P2 both activated PBMC (Fig. 5C). FBRL-P3 lacked the typical GAR/RGG sequence and it showed no PBMC activation (Fig. 5A, C). GAR1 contains a 43-AA GAR/RGG motif near its C-terminus (Fig. 5D). It was expressed as GAR1-HA, which strongly induced TNFα from PBMC (Fig. 5E). Among these 3 nucleolar alarmins, NCL was the strongest (Fig. 5F). NCL completely lost its alarmin activity upon GAR/RGG deletion (Fig. 3B), but the GAR/RGG-deleted FBRL mutant may still exhibit residual alarmin activity depending on the PBMC donors (Fig. 5B, F). TLR ligand contamination of the purified FBRL-HA and GAR1-HA was excluded as trypsin digestion completely inactivated both proteins (Supplementary Fig. S1C). Collectively, our data suggest that the GAR/RGG motif is an alarmin motif, which may predict more nuclear alarmins^44^.

Our preliminary data showed NCL, FBRL and HMGB1 release by UV-induced necrotic cells (Supplementary Fig. S6A)^29^. NCL purified from the culture was similar to TxNE-derived NCL in monocyte activation (Supplementary Fig. S6B), suggesting a common pathway by which these nuclear alarmins signal tissue injuries.

## Discussion

In live cells, the nucleolus is deeply embedded^45,46^, which excludes nucleolar antigens from B-cell recognition. When antigen-presenting cells (APC) phagocytose apoptotic cells, nuclear antigens are only expected to be exposed to endosomal TLRs. However, these TLRs (TLR3 and TLR7-9) generally lack recognition of protein alarmins^42,47^. Most protein alarmins activate surface TLRs (TLR2, TLR4, and TLR5)^42,48^. HMGB1 activates TLR2, TLR4 and TLR5^40,41^. Here, we report that NCL activates TLR2 and TLR4 (Supplementary Fig. S4). These surface TLRs generally lack access to endosomal alarmins.

While apoptotic cells maintain plasma membrane integrity and conceal nuclear antigens and alarmins, these intracellular components are variably exposed by necrotic cells^28,31^. We previously showed that the nucleolus was progressively exposed by necrotic cells^29^. We show here that NCL is released by UV-induced necrotic cells and the released NCL can activate monocytes (Supplementary Fig. S6). Like NCL, FBRL and HMGB1 were also released by these UV-irradiated necrotic cells (Supplementary Fig. S6).

Cell surface NCL presents a challenge to the above proposition as it is not hidden from cell surface TLRs and autoantibodies in patients. It is unclear whether cell surface NCL exposes its GAR/RGG motif in sufficient density to activate TLRs on neighboring cells. Soluble NCL activated monocytes much more weakly than plate-coated, multivalent NCL (Supplementary Fig. S5). Surface NCL may only activate TLRs when it is highly expressed on live cells but necrotic cell nucleoli naturally express abundant NCL^29^. Surface NCL could surge transiently, e.g., during serum-induced cell growth^22^, which could help recruit immune or stem cells^49,50^. Surface NCL undergoes extensive modifications, including phosphorylation and glycosylation^25,51^, which may also modify its alarmin activity.

The discovery of increasing nuclear proteins with alarmin activities suggests hierarchical signals associated with tissue injury that orchestrate multiple homeostatic and defense responses^52,53^. One physiological benefit could be the promotion of CD8 T cell immunity when DC capture necrotic cells caused by viral infections or malignancy^54–56^. However, autoimmunity can occur when necrotic cells breach immune surveillance^27,57^. For example, necrotic cells release HMGB1-associated nucleosomes that elicit ANA and cause lupus-like diseases^58^. During SLE disease flares, nuclear antigens surge in the blood, suggesting excessive necrotic cell death^59,60^. On the contrary, HMGB1 also dampens TLR-mediated macrophage activation to reduce the necrosis of these tissue scavengers^61^. Tissue inflammation also recruits complement, which is a self-limiting humoral scavenging system for effective tissue cleansing^34^. Through inducing tissue inflammation, alarmins can recruit diverse cell type to sites of tissue injury^62^, including stem cells, which are required for tissue repair^49,50^.

## Supplementary information

Supplementary materials
