# Supplementary material for: The GAR/RGG motif defines a family of nuclear alarmins
Source: Cell Death Dis. 2021 May 12;12(5):477. doi: 10.1038/s41419-021-03766-w (PMC8116331; doi:10.1038/s41419-021-03766-w)
Supplement: Supplementary file 1 — Supplementary materials [file 41419_2021_3766_MOESM1_ESM.docx]

Supplementary Materials for

**The GAR/RGG motif defines a family of nuclear alarmins**

Shan Wu, Boon Heng Dennis Teo, Seng Yin Kelly Wee, Junjie Chen, and Jinhua Lu*

*Correspondence to: miclujh@nus.edu.sg

Supplementary Figures

In this document, 6 figures are provided to show: 1) the lack of TLR contamination, 2) autocrine activation of monocytes by IL-1β, 3) optimal inhibitor st-2825 and Ac-YVAD concentrations, 4) NCL specificity for TLR2/4, 5) enhanced NCL stimulation of monocytes at multi-valency, 6) necrotic cells release NCL as an alarmin.


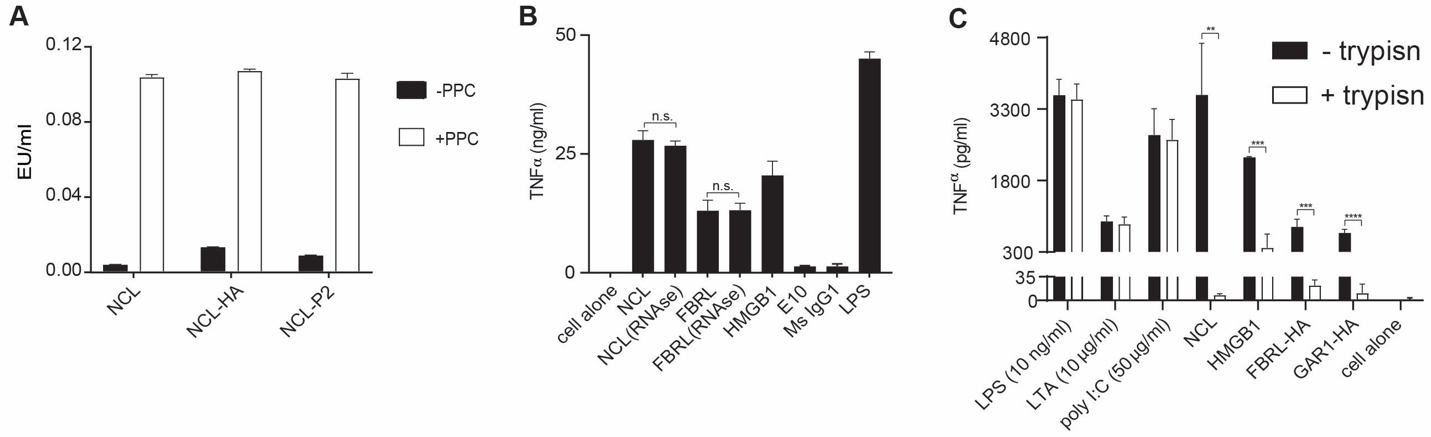


Fig. S1. **Inspection of TLR ligands in purified nuclear alarmins**.

(A) Purified NCL and NCL-HA (40 μg/ml) or the synthetic NCL-P2 peptide (200 μg/ml) in PBS were analyzed using the Limulus amoebocyte lysate assay. The assay was performed with or without adding product positive control (PPC, 0.1 EU/ml LPS) to the protein/peptide samples (+/-PPC). The activity of added LPS was mostly (>90%) recovered, showing the lack of LPS inhibition by the protein/peptide samples. Without added PPC, all samples exhibited < 0.5 EU/ml of LPS activity. (B) After coating with NCL and FBRL, plates were incubated for 1 hr at room temperature with RNase A (100 μg/ml in PBS) (Qiagen, Hilden, Germany, #19101). Plates were then washed to stimulate PBMC. As controls, NCL, FBRL, HMGB1, E10 and Ms IgG1 were coated to stimulate PBMC without prior RNase digestion. TNFα production was determined by ELISA. LPS (10 ng/ml) was added to PBMC as a positive control. (C) Purified proteins (40 μg/ml) were coated in 96-well plates (50 μl/well). After washing with PBS (200 μl/well), plates were incubated for 1 hr at 37^o^C with trypsin (Promega, Madison, WI, #V5280) at 50 μl/well (2 μg/ml in PBS). As a control, plates were not incubated with trypsin. PBMC (3x10^6^/ml) were incubated in these coated plates for 24 hr. As controls, LPS (200 ng/ml, 18 μl), LTA (200 μg/ml,18 μl) and poly I:C (1 mg/ml,18 μl) were pre-incubated with trypsin or PBS (9:1, v/v) at room temperature for 1 hr at 37^o^C. PBMC were cultured in blank wells to be stimulated for 24 hr with these trypsin-treated TLR ligands: LPS (10 ng/ml), LTA (10 μg/ml) and poly I:C (50 μg/ml). TNFα production was determined by ELISA. Experiments were performed in triplicates and presented as means ± SD. Statistics was performed by student t test. *p<0.05, **p<0.01, ***p<0.001, ****p<0.0001.


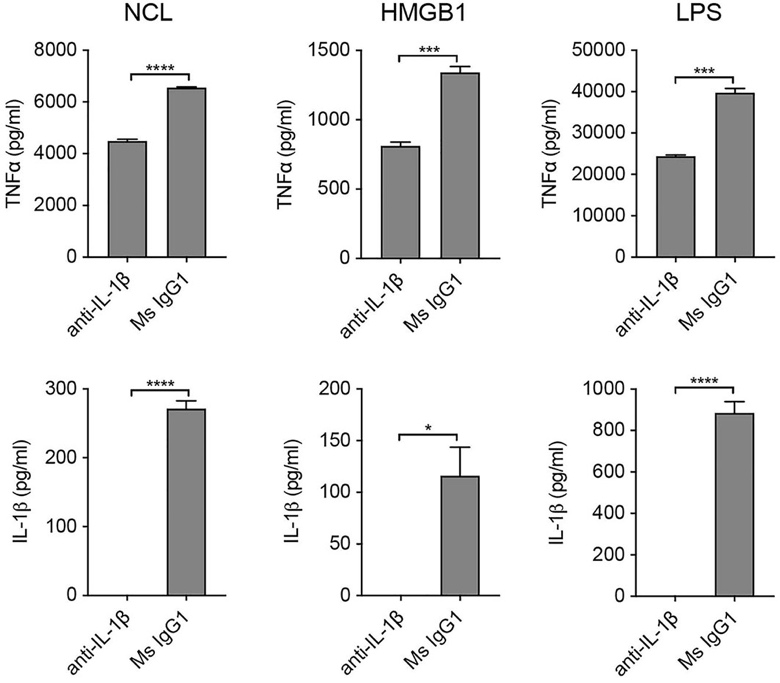


Fig S2. IL-1β-dependent TNFα induction by NCL, HMGB1 and LPS.

Monocytes stimulated with coated NCL or HMGB1 (40 μg/ml) or soluble LPS (10 ng/ml) in the presence of either a mouse IL-1β-blocking antibody or non-immune mouse IgG1 (10 μg/ml). After 24 hr, TNFα and IL-1β were determined in the media by ELISA. Experiments were performed in triplicates and presented as means ± SD. Statistics was performed by student t test. *p<0.05, **p<0.01, ***p<0.001, ****p<0.0001.


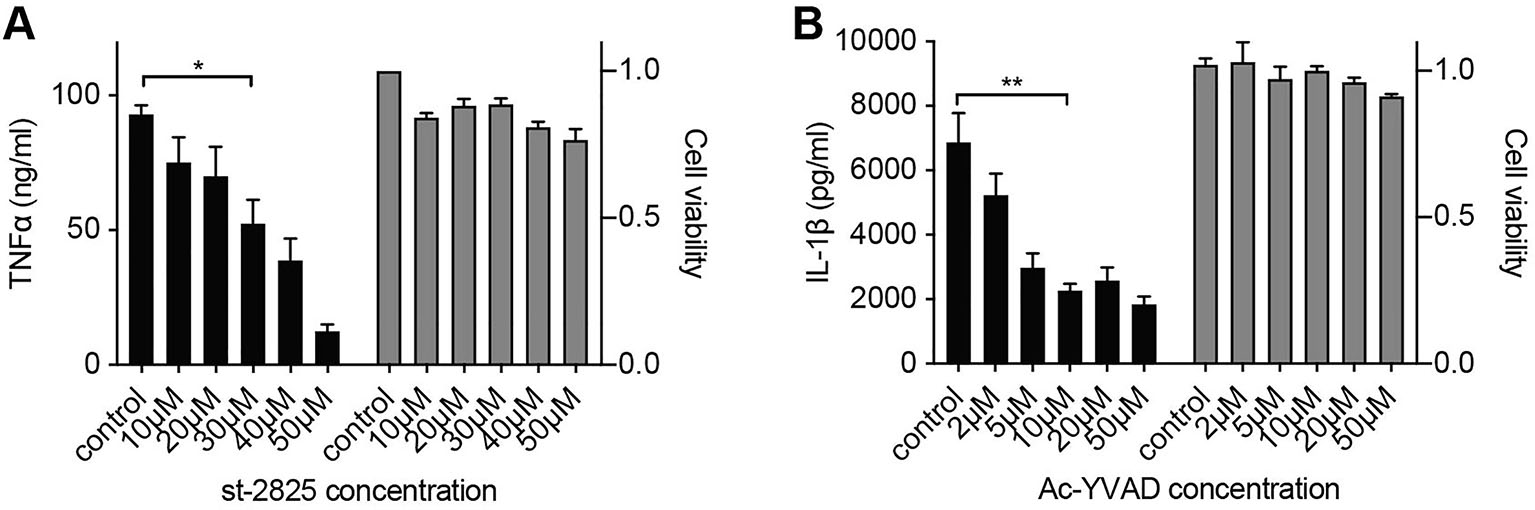


Fig S3. Titration of the MyD88 inhibitor st-2825 and caspase 1 inhibitor Ac-YAVD. Monocytes were pre-incubated with st-2825 (A) or Ac-YVAD (B) for 1 hr at different concentrations and then stimulated for 24 hr with LPS (10 ng/ml). TNFα and IL-1β production was measured by ELISA and cell viability was determined using the colourimetric MTS assay (Promega). Data was expressed as relative cell viability taking readings from untreated cell cultures (controls) as 1.0. Experiments were performed in triplicates and presented as means ± SD. Data was analyzed by student *t* test. * p < 0.05, ** p < 0.01.


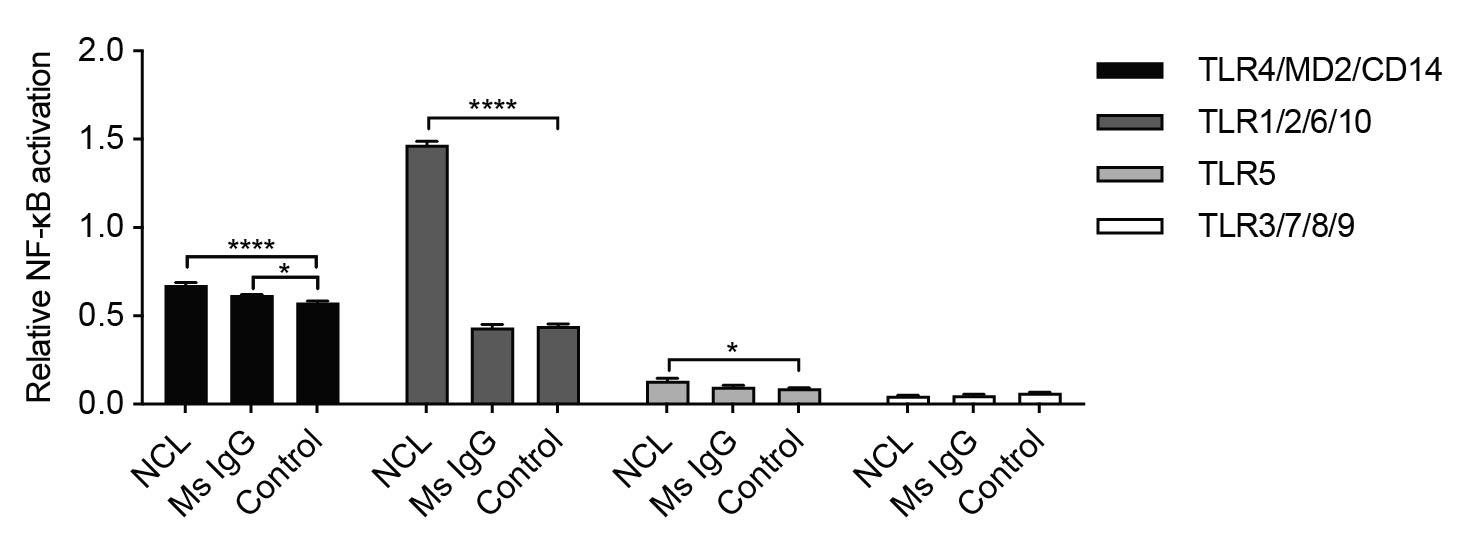


Fig S4. **TLR activation by NCL.**

HEK 293T cells were transfected with the p5xNFκB-Luc and pRL-CMV vectors and co-transfected with the TLR4/MD2/CD14, TLR2/1/6/10, TLR5, or TLR3/7/8/9 group of vectors. Each vector was used at 0.1 μg/well in 24-well plates. After 24 hr, cells were harvested and stimulated in plates coated with NCL or elution from immobilized mouse IgG1 (Ms IgG). Cells were stimulated for 24 hr and NFκB-mediated luciferase activity was determined in each well using the Dual Luciferase Reporter Assay System (Promega). Relative NFκB activation was derived by normalizing the firefly luciferase activity detected in each well to the *Renilla* luciferase activity. Triplicate experiments were performed and data were presented as mean ± SD. Control, transfected cells were cultured in wells without coating. Statistics was performed by one-way ANOVA. **** p < 0.0001; ** p < 0.01.


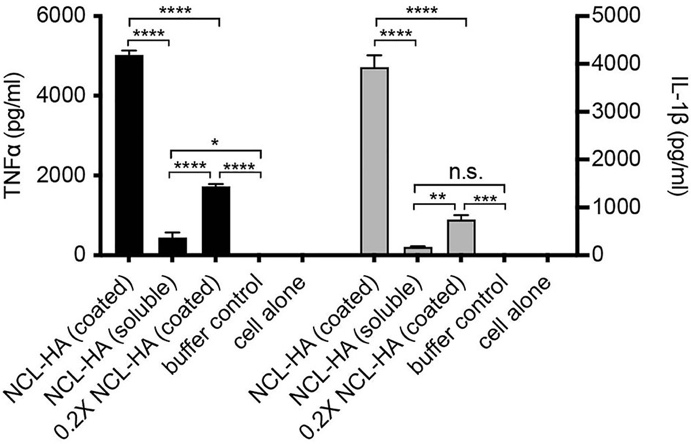


Fig S5. **Plate-coated NCL-HA activates monocytes more strongly than soluble NCL-HA.** Monocytes (1x10^5^/well) were cultured for 24 hr in plates which were either pre-coated with NCL-HA (40 μg/ml) or uncoated but added with soluble NCL-HA (40 μg/ml). Monocytes were also cultured in plates which were coated with NCL at 8 μg/ml (0.2x NCL-HA). Buffer control represents wells coated with buffer only. Cell alone, wells that were not coated. TNF**α** and IL-1**β** in the media were determined by ELISA. Experiments were performed in triplicates and presented as mean ± SD. Statistics was performed by one-way ANOVA. * p < 0.05, ** p < 0.01. *** p < 0.001, **** p < 0.0001, n.s., not significant.


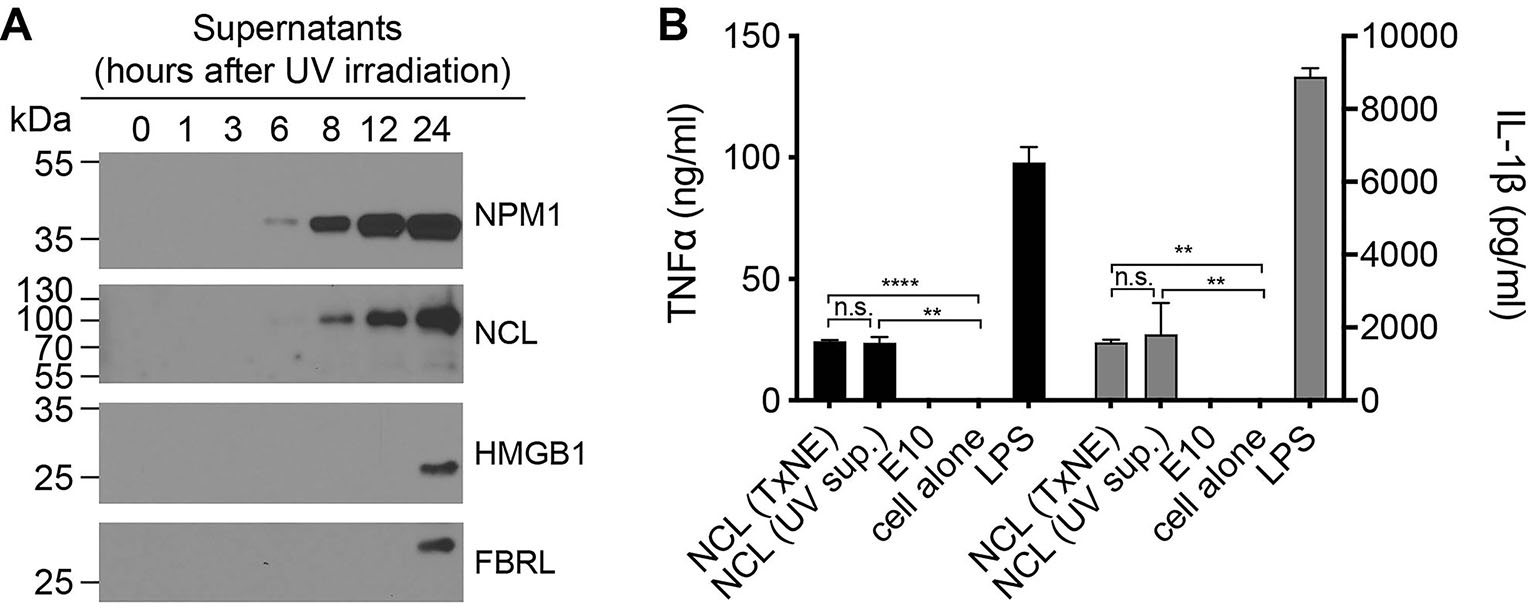


Fig S6. **NCL released by UV-irradiated dead cells activates monocytes.**

(A) HeLa cells were cultured in 150-mm dishes and UV-irradiated in serum-free media as previously described ^1^. Cells were cultured for 0-24 hr and media were harvested either immediately (0 hr) or after 1, 3, 6, 8, 12 or 24 hr. Media were passed through 0.22-μm filters and analyzed by SDS-PAGE (12.5% (w/v)) and Western blotting for the presence of NCL and three other nuclear proteins. NPM1, nucleophosmin-1; FBRL, fibrillarin. (B) NCL was affinity-purified from the media harvested from HeLa cells 24 hr after UV irradiation (24 hr) and compared with NCL purified from TxNE in monocyte activation. Proteins were coated on the plates (40 μg/ml) to stimulate monocytes. As controls, plates were coated with the protein-free E10 fraction. As a positive control, monocytes were stimulated with LPS in uncoated wells. After 24 hr, TNF**α** and IL-1**β** production was determined by ELISA. Experiments were performed in triplicates and presented as mean ± SD. Statistics was performed by one-way ANOVA. ** p < 0.01, **** p < 0.0001, ns: not significant.

1. Cai Y, Teo BH, Yeo JG & Lu J. C1q protein binds to the apoptotic nucleolus and causes C1 protease degradation of nucleolar proteins. *J Biol Chem* **290,** 22570-22580 (2015).
